# Outcomes and complications of different approaches for 1–2 cm upper tract stones in the paediatric population: bicentric retrospective analysis

**DOI:** 10.1007/s00383-026-06372-z

**Published:** 2026-04-15

**Authors:** Martina Bruniera, Giovanni Basso, Simone Botti, Martina Grossele, Michele Gnech, Alfredo Berrettini, Paolo Beltrami, Giovanni Montini, Francesca Taroni, Maria Cristina Mancuso, Enrico Vidal, Maria Sangermano, Germana Longo, Fabrizio Dal Moro, Alessandro Morlacco

**Affiliations:** 1 Urology Unit, Padova University, Padua, Italy; 2 Paediatric Urology Unit, Padova University, Padua, Italy; 3 Paediatric Nephrology Unit, Fondazione IRCCS Ca’ Granda Ospedale Maggiore Policlinico di Milano, Milan, Italy; 4 Paediatric Nephrology Unit, Azienda Ospedale Università Padova, Padua, Italy; 5 Endourology Unit, Azienda Ospedale Università Padova, Padua, Italy

**Keywords:** Paediatric urolithiasis, Renal stones, SWL, URS/RIRS, Mini-PCNL, Paediatric surgery

## Abstract

**Purpose:**

To evaluate outcomes and complications of different surgical approaches for 10–20 mm internal stones in children, based on bicentric real-world experience.

**Materials and methods:**

We retrospectively analysed 96 patients treated between 2009 and 2022 at two tertiary referral centres. Data included demographics, stone characteristics, treatment (extracorporeal shockwave lithotripsy -SWL, retrograde surgery -RIRS, percutaneous nephrolithotomy – PCNL, surgery), complications, and stone-free rates. The primary outcome was stone clearance; complications were graded according to the Clavien-Dindo classification. Categorical variables were compared using Chi-square of Fisher’s exact test.

**Results:**

Median age was 61 months (IQR 25–105). Nineteen patients (19.8%) had associated urological conditions. 50% had multiple stones. Treatments included SWL (17.7%), URS/RIRS (46.9%), PCNL (19.8%), and surgery (8.3%). Intraoperative complications occurred in 4.2%, early postoperative in 12.5%, and late complications in 11.5%. Stone clearance after the first procedure was achieved in 47.9%. Clearance rates were 58.8% for SWL, 47.8% for URS/RIRS, and 52.6% for PCNL. No statistically significant differences were observed in stone clearance or complication rates among treatments.

**Conclusions:**

All treatments demonstrated safety and efficacy for 10–20 mm pediatric intrarenal stones. Individualized treatment planning, considering stone burden and patient-specific factors, remains essential to optimize outcomes.

## Introduction

 Paediatric urolithiasis (UL) is an increasing clinical challenge with rising incidence and substantial recurrence rates [1–5]. While surgical management strategies for stones < 10 mm and > 20 mm are relatively well-defined, treatment of stones measuring 10–20 mm remains controversial. Beyond the acute management for obstructive stones, UL in childhood has important long-term implications, including risks of chronic kidney disease and metabolic disorders, underscoring the need for effective early intervention.

Current guidelines from the European Society for Paediatric Urology (ESPU) and the American Urological Association (AUA) recognise extracorporeal shockwave lithotripsy (SWL), ureteroscopy (URS)/retrograde intrarenal surgery (RIRS), and percutaneous nephrolithotomy (PCNL) as viable options for this intermediate stone size. However, recommendations frequently overlap and are based on limited or low-quality evidence, leading to significant variability in clinical practice [6, 7].

Our study aims to compare stone clearance and complication rates of different surgical modalities for 10–20 mm intrarenal stones in children across two tertiary centres. We first describe overall treatment outcomes and then examine potential differences among modalities to inform strategies for optimizing clinical management in this patient population.

## Materials and methods

We conducted a bicentric retrospective study including 96 patients with intrarenal stones measuring 10–20 mm, treated between 2009 and 2022 at two tertiary paediatric urology centres. Procedures were performed by expert endourologists, with one primary surgeon per centre. Patients with incomplete data or who received initial treatment outside these institutions were excluded. Eighty-nine patients underwent a surgical approach, while 7 were successfully managed conservatively with medical therapy and active surveillance and were excluded from comparative statistical analyses.

Collected variables included demographics, age at initial intervention, presence of upper urinary tract anomalies, and history of prior surgeries and stone characteristics (number, size, and location).

**Fig. 1:**
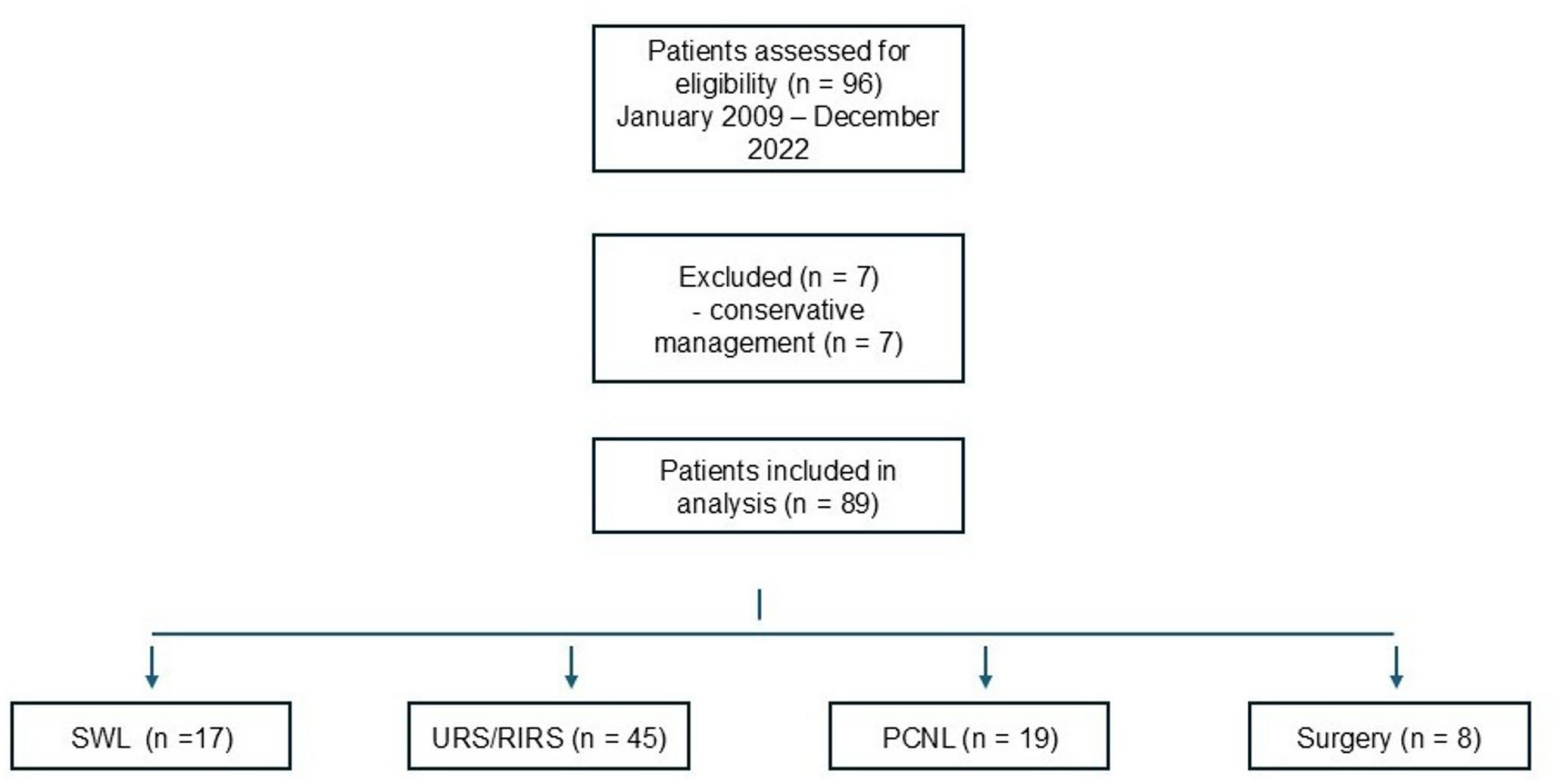
Flow diagram of patient selection and treatment allocation

Treatment selection was individualized based on multidisciplinary assessment and shared decision-making involving patients and families. Patients with concomitant anatomical abnormalities were treated according to current guidelines with open and robotic surgeries —open surgery primarily reserved for the youngest patients Figure 1.

### Surgical procedures

Shock wave lithotripsy (SWL) was performed using 2000–3000 shockwaves per session (60–80 shocks per minute) at power settings of 25–50% (20–40 mJ), general anaesthesia for younger patients.

Ureteral or renal retrograde surgery (URS/RIRS) employed flexible (7.5–9.5 Fr) or rigid scopes (6–8 Fr) pediatric ureteroscopes, with Holmium: YAG laser energy set at 0.5–1 J and frequencies above 8 Hz.

Percutaneous nephrolithotomy (PCNL) was performed via 11–22 Fr access, using ultrasonic and pneumatic lithotripsy devices, with initial access established collaboratively between interventional radiologists and urologists.

### Outcomes

Primary outcome was stone clearance, defined as absence of residual stones on imaging at first follow-up performed within 3 months from surgery and with ultrasound and/or X-ray. We considered residual fragments even if asymptomatic and not requiring re-intervention, was recorded as residual stone.

Complications were classified as intraoperative, early (pre-discharge), or late (within 90 days after discharge), and graded according to the Clavien Dindo classification.

Follow-up included clinical assessment and ultrasound imaging within three months post-discharge, followed by combined urological and nephrological evaluations every six months.

### Statistical analysis

Statistical analysis was performed using SPSS v26. Continuous variables are reported as medians with interquartile range (IQR), categorical variables as counts and percentages. Comparisons among the four treatment groups were performed using Chi-square or Fisher’s exact tests for categorical variables. A p-value < 0.05 was considered statistically significant and confidence interval (CI) of 95%, in case of significance appropriate post-hoc analyses was performed.

### Ethics statement

This study was retrospective and observational in nature. According to national regulations and institutional policies, formal approval by the local Ethics Committee was not required for retrospective studies based on anonymized data. The study was conducted in accordance with the Declaration of Helsinki.

## Results

A total of 96 patients were included, 89 (92.7%) underwent surgical intervention, while 7 (7.3%) were managed conservatively and reported descriptively only. The surgical cohort included 51 (53.1%) males, with a median age at treatment of 61 months (IQR 25–105 months). Baseline characteristics are summarized in Table 1.

**Table 1:** Categorical variables

Variable	*n*	Percentage (%)
*Sex*		
Male	51	53.1
Female	45	46.9
*Preoperative anatomical abnormalities*		
Vesicoureteral reflux	5	5.2
Hydronephrosis	2	2.1
Megaurether	1	1
Duplex kidney	2	2.1
Pyeloureteral disease	7	7.3
Horseshoe kidney	1	1
Posterior urethral valve	1	1
*Stone number*		
One	48	50
> One	48	50
*Stone localization*		
Pelvi	31	32.3
Calyx	27	28.1
Both	38	39.6
*Further procedure required*		
Yes	48	53.9
No	41	46.1
*Surgical approach*		
SWL	17	19.1
URS/RIRS	45	50.6
PCNL	19	21.3
Surgery	8	9.0
*Median age*	61 months (IQR 25–105 months)	
*Median stone size*	15 mm (IQR 12–18 mm)	

*VUR* vesicoureteral reflux, *PUV* posterior urethral valve, *SWL* extracorporeal shockwave lithotripsy, *URS/RIRS* ureteral or renal retrograde surgery, *PCNL* percutaneous nephrolithotomy

Nineteen (19.8%) patients presented with associated urological disorders, mostly vesico-ureteral-reflux (VUR) and ureteropelvic junction obstruction (UPJ). Four patients had previously undergone pyeloplasty. Multiple stones were presents in 48 (50%) patients. Stones localized in both the renal pelvis and calyces in 38 (39.6%) patients, in the pelvis only in 31 (32.3%), and in calyces in the remainder. Median size of stones was 15 mm (IQR 12–18 mm). Surgical modalities included 17 (19.1%) SWL, 45 (50.6%) URS/RIRS, 19 (21.3%) PCNL, 8 (9.0%) open or robotic surgery, all of whom had concomitant urological anomalies.

### Complications

Intraoperative complications occurred in 4 (4.4%) patients, mainly bleeding or urine leakage during endourological procedures. Early complications were observed in 12 (13.5%) patients, most commonly fever (*n* = 11), equally distributed, 4 PCNL, 4 RIRS, 2 SWL and 1 after surgery. Late complications were reported in 11 (12.9%) patients, especially fever that required oral antibiotic treatment, Table 2.

**Table 2:** Complications divided into intraoperative, early and late

Complication	SWL – (95% CI)	URS/RIRS – (95% CI)	PCNL – (95% CI)	Surgery – (95% CI)	Tot − (95% CI)	*p*-value
*Intraoperative*						
Yes	0 – (0.0–18.4)	2 (4.5%) – (1.2–14.8)	2 (10.5%) – (2.9–31.4)	0 – (0.0–32.4)	4 (4.4%) – (1.8–10.9)	0,314
No	17 (100%)	43 (95.5%)	17 (89.5%)	8 (100%)	85 (95.6%)	
*Early*						
Yes	2 (11.8%) – (3.3–34.3)	4 (8.9%) – (3.5–20.7)	4 (21.1%) – (8.5–43.3)	2 (25.0%) – (7.10–59.1)	12 (13.5%) – (7.1–59.9)	0,717
No	15 (88.2%)	41 (91.1%)	15 (78.9%)	6 (75.0%)	77 (86.5%)	
*Late*						
Yes	1 (5.9%) – (1.0–27.0)	7 (15.6%) – (7.8–28.8)	1 (5.3%) – (0.9–24.6)	2 (25.0%) – (7.1–59.1)	11 (12.9%) – (7.0–21.0)	0,433
No	16 (94.1%)	38 (84.4%)	18 (94.7%)	6 (75.0%)	76 (87.1%)	
*TOT*	3 (17.6%)	13 (28.9%)	7 (36.8%)	4 (50.0%)	27 (30.3%)	

*SWL* extracorporeal shockwave lithotripsy, *URS/RIRS* ureteral or renal retrograde surgery, *PCNL* percutaneous nephrolithotomy

When comparing patients with and without complications each treatment modality, no statistically significant association was observed between the type of procedure and the occurrence of intraoperative complications (Fisher’s exact test *p* = 0.314), early complications (Fisher’s exact test *p* = 0.717) and late complications (Fisher’s exact test *p* = 0.4330). Overall intraoperative complication rate was 4 (4.4%) – (95% CI 1.8% – 10.9%), early complication rate 12 (13.5%) – (95% CI 7.1% – 59.9%), late complication rate 11(12.9%) – (95% CI 7.0% – 21.0%).

Overall complications occurred in higher rate in patients with anatomical malformations compared to those without malformations (*p* = 0.001).

The presence of multiple stones was not significantly associated with postoperative complications (*p* = 0.568). Likewise, stone localization in lower pole (*p* = 0.375) and stone size > 15 mm (*p* = 0.135) were not significantly associated with complication rates, Table 3.

**Table 3:** Subgroup analyses for complications and stone clearance

Variable	Complications OR (95% CI)	*P* value	Stone clearance OR (95% CI)	*P* value
Anatomical malfourmations	0.765 (0.587–0.995)	0.001	1.126 (0.388–3.625)	0.519
Multiple	0.723 (0.097–5.394)	0.568	0.379 (0.156–0.955)	0.026
Lower pole	0.378 (0.038–3.796)	0.375	1.654 (0.686–3.955)	0.185
Size	0.176 (0.017–1.772)	0.135	2.262 (0.899–5.692)	0.063

**Table 4:** Stone clearance after 1st procedure

SFR	SWL – (95% CI)	URS/RIRS – (95% CI)	PCNL – (95% CI)	Surgery – (95% CI)	Tot - (95% CI)	*P* value
Yes	10 (58.8%) – (36.0–78.4)	23 (51.1%) – (37.0–65.0)	10 (52.6%) – (31.7–72.7)	5 (62.5%) – (30.6–86.3)	48 (53.9%) – (43.6–64.0)	0,948
No	7 (41.2%)	22 (48.9%)	9 (47.4%)	3 (37.5%)	41 (46.1%)	

*SWL* extracorporeal shockwave lithotripsy, *URS/RIRS* ureteral or renal retrograde surgery, *PCNL* percutaneous nephrolithotomy

### Stone clearance

Complete stone clearance during the procedure and at follow up was achieved after the first procedure in 48 (53.9%) patients. By treatment modality 10 (58.8%) SWL, 23 (51.1%) URS/RIRS, 10 (52.6%) PCNL and 4 (62.5%) surgery, one underwent nephrectomy, Table 4.

When comparing patients with and without stone clearance after the first procedure, no statistical association was observed between treatment modality and stone clearance achievement (*p* = 0.948).

Two (4.9%) patients required an additional ESWL session, 13 (31.7%) underwent URS/RIRS, 5 (12.2%) PCNL and 2 (4.9%) surgical procedures to complete stone clearance. Overall, 5 (12.2%) patients required multiples interventions to achieve stone clearance as defined, and 9 (21.9%) was treated on the contralateral side. However, detailed information regarding residual is missing.

The presence of multiple stones was significantly associated with stone clearances rates (*p* = 0.026).

In contrast, urinary malformations (*p* = 0.519), lower pole stone localization (*p* = 0.185), and stone size > 15 mm (*p* = 0.063) were not significantly associated with stone clearance, Table 3.

## Discussion

In this cohort of children with 10–20 mm renal stones, no single surgical approach demonstrated clear superiority in terms of stone clearance or complication rates. Although SWL and URS/RIRS achieved comparable success rates; these findings should be interpreted with caution given the heterogenicity of the population and retrospective nature of the study.

Literature reports SWL success rates between 59% and 94% particularly favourable for small stones, pelvic or upper calyx localization, and female patients. In our cohort SWL achieved a stone clearance within the reported range. However, parameters such as radiation exposure, operative time were not directly measured in this study and therefore no conclusions can be drawn regarding potential advantages [8–11].

Although URS/RIRS typically demonstrates higher success rates (87–100%), yet our cohort showed lower results, comparable to SWL. A contributing factor was the frequent need for DJ placement due to narrow ureters, which we considered a first-procedure failure, unlike other studies that routinely pre-stented. Furthermore, we analysed two high volume tertiary centre we have a huge number of complex cases and the high prevalence of multiple stones and anatomical anomalies likely to contributed to lower first-session clearance. Moreover, the impact of multiple stone is associated with a higher risk of not achieve the stone clearance after first operation.

PCNL achieves clearance rates 80.6% and 92.4%, regardless of age, but is often perceived as more invasive. Literature shows no major differences in clearance or complications between mini- and micro-PCNL [9, 12], supporting both as alternatives to SWL [11, 13]. Abuelunga et al. found similar efficacy between URS/RIRS and mini-PCNL, with shorter hospitalization and less radiation for URS/RIRS [14]. Our findings are also consistent with those of Unal et al., who reported comparable outcomes across techniques, except for lower pole stones, which favour PCNL [15]. While radiation exposure is a recognized concern, this was mitigated in our centres by combining fluoroscopic and ultrasound guidance, although radiation does was not quantitatively assessed.

Major surgical approaches -open or robotic- remain reserved for complex cases, including children with anatomical abnormalities or failed endourological procedures [16]. While effective, these procedures require longer anaesthesia, pain control.

In our cohort these techniques were selectively adopted in patients with concomitant urological anomalies, which limits direct comparison with minimally invasive approaches.

Several authors have proposed treatment algorithms based on stone size and location. A review from 2018 proposed a different approach for renal stones according to their position in the lower calyx. SWL as the first line and URS/RIRS or PCNL as the second line, except for lower calyx stones where PCNL could be selected as first line therapy [17]. Similarly, Samotyjek et al. suggested treating stones in the upper urinary tract < 20 mm with SWL and > 15 mm with PCNL, while preferring PCNL for stones > 10 mm in lower urinary tract [18]. These recommendations align with our observation that stone location and burden are key factors influencing treatment outcomes. Few studies have reported, with statistical significance, that SWL is a less effective technique, with insufficient data on long-term kidney damage [19].

Overall, our results support an individualized approach to pediatric renal stones 10–20 mm, in which treatment selection should be guided by stone size, location, multiplicity, anatomical considerations, and patient age, rather than by an assumption of equivalence among modalities. We observed a higher number of complications in patients with anatomical malformations, highlighting that complex cases need to be carefully evaluated and, when appropriate, centralized in centres capable of managing potential complications. Additionally, we found that patients with multiple stones underwent more procedures, which is important for preoperative counselling with both, surgeon and family.

### Limitations

This study has several limitations. Its retrospective design and clinically driven treatment allocation introduce an inherent risk of selection bias. The long inclusion period (2009–2022) spans substantial technological evolution in endourology, including advances in miniaturized instruments, flexible ureterorenoscopes, which may have influenced outcomes, and the decision make process supporting the choice of URS/RIRS. In addition, the small sample size within each group, limits statistical power and procedures definitive comparative conclusions. Larger prospective, standardized, and ideally randomized studies are required to better define optimal treatments strategies for pediatric intrarenal stones.

Few studies directly compare stone location and clearance across procedures, and current recommendations may evolve with new technology. Larger prospective randomized studies are needed to confirm these results.

## Conclusion

In this biocentric cohort, no statistically significant difference in stone clearance or complication rates were observed among the evaluated treatment modalities for pediatric renal stones, measuring 10–20 mm. Treatment selection supports an individualized approach to surgical planning rather than the assumption of equivalence among techniques considering that in case of anatomical malformation we have a higher number of complications and in case of multiple stone a higher number of treatment to achieve the stone clearance.

These findings should be interpreted considering study’s limitations, larger prospective studies are required to confirm the observations and to further refine treatment strategies in this patient population.

## Data Availability

No datasets were generated or analysed during the current study.
